# Interleukin-33 enhances ASIC currents in mouse dorsal root ganglion neurons

**DOI:** 10.1016/j.jbc.2026.113398

**Published:** 2026-08-07

**Authors:** Ting-Ting Liu, Xue-Mei Li, Chun-Yu Qiu, Wang-Ping Hu

**Affiliations:** School of Basic Medical Sciences, Xianning Medical College, Hubei University of Science and Technology, Xianning, Hubei, PR China

**Keywords:** interleukin-33, acid-sensing ion channel, current, dorsal root ganglion neuron, mechanical hyperalgesia

## Abstract

Cytokine interleukin-33 (IL-33) signaling in primary sensory neurons plays a crucial role in pain. However, the underlying molecular mechanisms remain poorly understood. Therefore, we investigated whether IL-33 signaling affects ion channels in nociceptive dorsal root ganglion (DRG) neurons. Herein, we reported that the application of IL-33 enhanced the electrophysiological activity of acid-sensing ion channels (ASICs). IL-33 dose-dependently increased acid-evoked ASIC currents in mouse DRG neurons. IL-33 enhanced the maximum responses of ASICs, whereas the sensitivity to acidic stimuli remained unaffected. This IL-33-induced enhancement of ASIC currents was dependent on suppression of the tumorigenicity 2 (ST2) receptors. The enhancing effect of IL-33 on ASIC currents was prevented by the p38 mitogen-activated protein kinase inhibitor SB202190, but not by the ERK inhibitor U0126 or the JNK inhibitor SP600125, indicating the effect was p38-dependent. Moreover, IL-33 potentiated the action potential triggered by acidic stimuli. Finally, ASIC3-deficient mice displayed attenuated mechanical hyperalgesia induced by intraplantar or intramuscular injection of IL-33. Our findings revealed that IL-33 enhanced ASIC function *via* ST2 and the intracellular p38 signaling pathway, which might provide a promising therapeutic approach for pain treatment by targeting IL-33/ST2 signaling.

Interleukin-33 (IL-33) is a cytokine of the IL-1 superfamily. IL-33 located in the nucleus *in vivo* under normal physiological state and is rapidly released into the extracellular environment after cell damage and stress (1, 2, 3). IL-33 can elicit a variety of biological functions by binding to two different endogenous suppressors of tumorigenicity 2 (ST2) receptor isoforms, soluble ST2 (sST2) and transmembrane ST2 (1, 4). The transmembrane ST2 mainly mediates the actions of IL-33, while sST2 limits IL-33 activity as a decoy receptor (5, 6). It has demonstrated that IL-33 can directly activate primary sensory neurons through ST2 expressed in these neurons (7, 8, 9). Evidence has revealed that IL-33/ST2 signaling plays a crucial role in nociceptive behaviors (10). Intraplantar or intraarticular injection of exogenous IL-33 in normal mice produces nociception, while IL-33 failed to evoke similar nociceptive behaviors in mice lacking ST2 (11, 12, 13). Studies have shown that IL-33/ST2 signaling is implicated in various models of inflammatory and neuropathic pain (14). Upregulation of IL-33 and ST2 has been detected in these pain model animals, and an anti-IL-33 neutralizing antibody can effectively alleviate nociceptive behaviors by blocking IL-33 (15, 16). For example, increased IL-33 levels have observed in the hind paw after a complete Freund's adjuvant injection (17). The neutralization of IL-33 in the dorsal root ganglion (DRG) with an anti-IL-33 neutralizing antibody alleviated mechanical allodynia in a sciatic nerve injury model (18). Therefore, strategies targeting IL-33/ST2 signaling have shown a favorable analgesic profile (19). Nevertheless, the molecular mechanisms underlying IL-33/ST2 signaling in peripheral nociceptive responses remain poorly understood. It has shown that ST2 is functionally expressed in primary nociceptive neurons, including DRG and trigeminal ganglion neurons (7, 20). Recent studies report that IL-33 inhibits type A potassium channels and increases neuron excitability in mouse DRG neurons through ST2 (17). IL-33 potentiates TRPA1 function in trigeminal ganglion neurons (20). Primary nociceptive neurons are critical structures in pain transduction and modulation, as these neurons express multiple ion channels, including voltage-gated and ligand-gated ion channels (21). Those pain-associated ion channels detect noxious stimuli and play a critical role in pain (22). However, the role of IL-33/ST2 signaling on the ion channels localized on primary nociceptive neurons is still poorly understood.

Acid-sensing ion channels (ASICs) are a group of proton-gated ion channels that are expressed in primary sensory neurons (23, 24, 25). Molecular cloning has revealed that four genes encode at least six ASIC subunits (ASIC1a, −1b, −2a, −2b, −3, and −4), which form functional ion channels in either homotrimeric or heterotrimeric complexes (26, 27). Different ASIC complexes have different pH sensitivities, allowing them to detect a wide range of pH levels (28). In all ASIC complexes, those channel complexes containing ASIC3 are sensitive to slight extracellular acidosis and are considered critical pH sensors. Tissue acidification occurs in a variety of pain conditions. ASICs and transient receptor potential vanilloid type 1 (TRPV1) expressed in small-diameter DRG neurons are very important extracellular acid sensors, contributing to acid-induced nociception within the physiological pH range, with below pH 7.3 for ASICs and below pH 6.0 for TRPV1 (29, 30). The acid-induced pain is alleviated by the non-selective ASIC inhibitor amiloride, indicating the involvement of ASICs (31, 32). Among all ASIC subunits, the ASIC3 subunit is the most abundantly expressed in primary sensory neurons (33). The expression levels of ASIC3 in DRG neurons are increased under conditions of nerve injury and inflammation (33). Subcutaneous injection of acidic solutions into animal hind paws induces nociceptive behaviors that were completely inhibited by the ASIC3 blocker APETx2 (33). ASIC3-deficient mice also exhibit attenuated nociceptive behaviors (34, 35). So ASIC3 has emerged as a major player in pain during tissue acidification (36).

Sensitization of primary afferents is essential for the development of pain. Since both IL-33/ST2 signaling and ASICs are present in primary sensory neurons and are also involved in pain processing, the purpose of this study was to investigate whether there is a functional link between them in peripheral nociceptive processing. Herein, we reported that IL-33 binding to ST2 increased ASIC-mediated currents in mouse DRG neurons *via* a p38 mitogen-activated protein kinase (MAPK)-dependent pathway. Behaviorally, we observed that ASIC3 knock-out (KO) mice displayed attenuated mechanical hyperalgesia induced by intraplantar or intramuscular injection of IL-33 compared to wild-type (WT) littermates.

## Results

### IL-33 enhances ASIC currents in mouse DRG neurons

**Figure 1:**
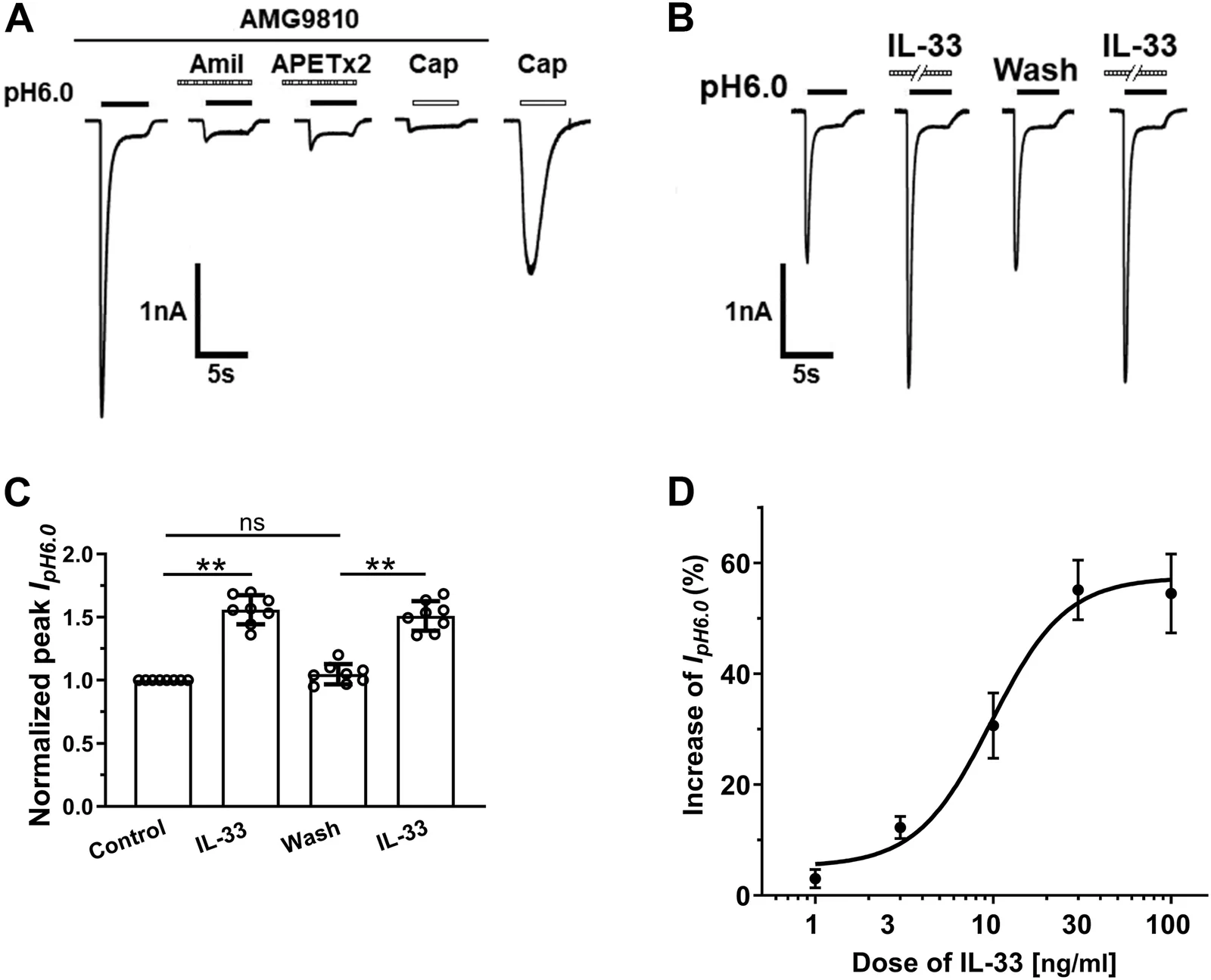
**Enhancement of ASIC currents by IL-33 in mouse DRG neurons.***A*, exposure of a DRG neuron to acidic solution at pH 6.0 caused a rapid inward current (I_pH6.0_). The acid-evoked current was blocked by amiloride (Amil, 100 μM) and APETx2 (2 μM). In the recorded cell, capsaicin (Cap, 100 nM) induced no membrane currents in the presence of 5 μM AMG9810, and an inward current in the absence of AMG9810. The membrane potential of the cell was clamped at −60 mV. The representative current traces in (*B*) and the bar graph in (*C*) show that the amplitude of I_pH6.0_ was enhanced by pre-application of 30 ng/ml IL-33 for 5 min, recovered after washout of IL-33 for 5 min, and was enhanced again when IL-33 was applied again in the same manner. ∗∗*p* < 0.01, One-way ANOVA followed by Tukey's *post hoc* test. n = 8 cells from 6 mice in each column. *D*, the graph shows that the dose-effect curve of IL-33 on I_pH6.0_ with an EC_50_ value of 9.7 ± 1.3 ng/ml. Each point represents the means ± SD of 8 to 9 cells from 5 to 7 mice.

In the majority of recorded DRG neurons (70.6%, 12/17), application of acidic solution at pH 6.0 for 5 s could cause a rapid inward current (I_pH6.0_), even if addition of AMG9810 (5 μM) in external solution (Fig. 1*A*). We observed that I_pH6.0_ was blocked by the broad-spectrum ASIC channel blocker amiloride (10 μM) and ASIC3 blocker APETx2 (2 μM). In all DRG cells sensitive to acidic stimuli, the TRPV1 agonist capsaicin (100 nM) failed to elicit any membrane response in the presence of AMG9810; in contrast, it evoked an inward current in the majority of recorded DRG cells (83.3%, 10/12) after washout of AMG9810. Therefore, these acid-evoked currents were identified as ASIC currents or ASIC3-like currents. In all subsequent cell experiments, we used APETx2 to identify all acid-induced currents. Only those currents were considered to be inhibited by APETx2 if their peak amplitude dropped by more than 90%. To specifically study ASIC3-like currents, only the currents inhibited by APETx2 were considered.

In some DRG neurons sensitive to acidic stimuli (66.7%, 8/12), we found that the I_pH6.0_ amplitude was increased when these cells were pretreated with IL-33 (30 ng/ml, equivalent to 1.7 nM) for 5 min prior to the subsequent current recording (Fig. 1*B*). The I_pH6.0_ amplitude recovered to control levels after washout of IL-33 for 5 min. In the present study, a cutoff value was considered to indicate a response of neurons to IL-33 only when the increase in I_pH6.0_ amplitude exceeded 6%; otherwise, it was considered an unresponsive cell. In the following experiments, mean values of current increase by IL-33 were calculated based on the IL-33-responsive cells. Figure 1*C* shows that the mean amplitude of I_pH6.0_ increased 55.9 ± 4.1% in DRG cells treated with 30 ng/ml IL-33 compared to the control (*p* < 0.01, one-way ANOVA followed by Tukey's *post hoc* test, n = 8), and subsequently recovered after washout of IL-33. Then, we applied IL-33 again to the same cell for 5 min, which also produced a similar enhancement of ASIC currents, indicating that the effect of IL-33 can be reproduced (Fig. 1B and C). The subsequent blocker experiments were conducted following this pattern. We first investigated whether the IL-33-mediated enhancement of I_pH6.0_ was dose-dependent. Figure 1*D* shows the dose-effect curve of IL-33 on I_pH6.0_ amplitude. The half-maximal effective dose (EC_50_) value of IL-33 was 9.7 ± 1.3 ng/ml. The results indicated that IL-33 increased ASIC currents in a dose-dependent manner.

**Figure 2:**
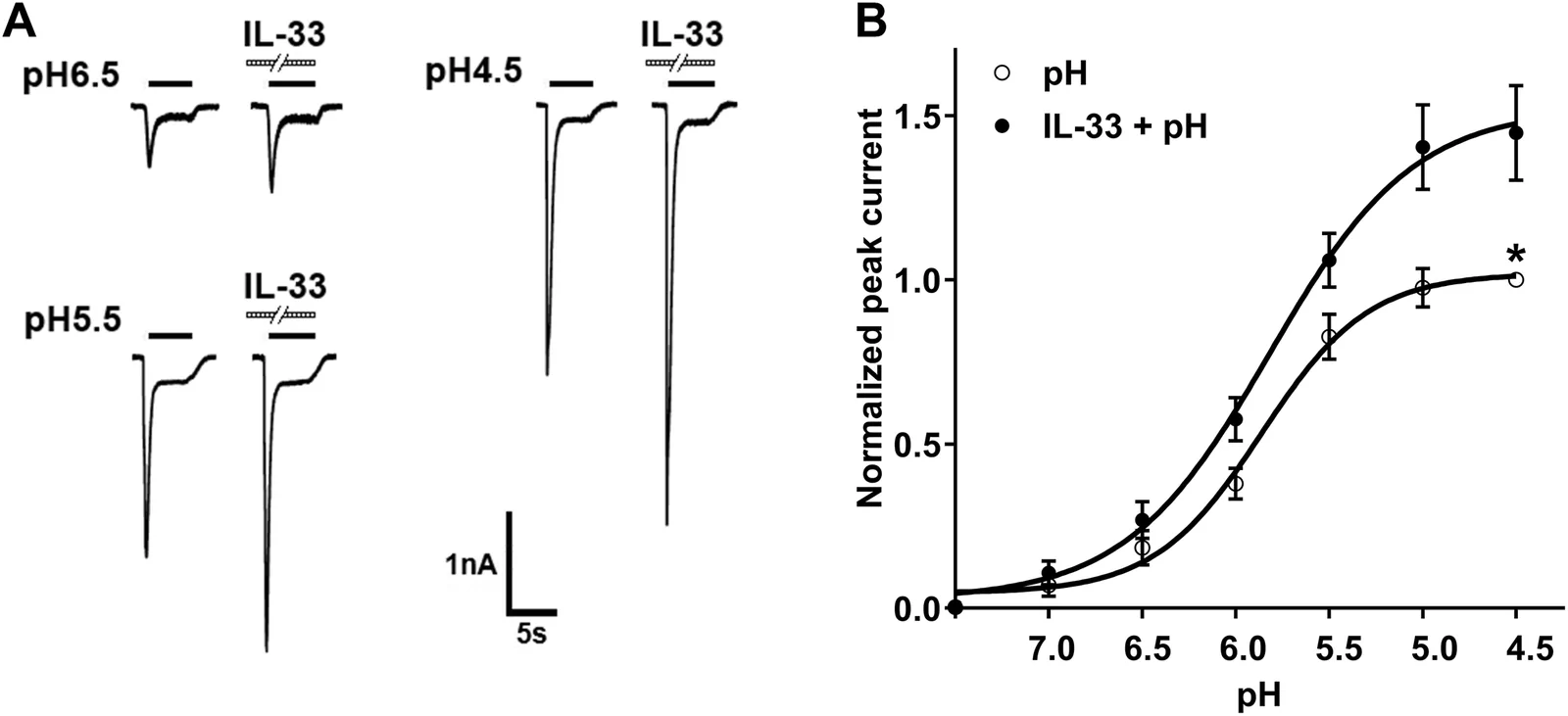
**Effects of IL-33 on the concentration–response curve for acid stimuli.***A*, representative current traces show that IL-33 pretreatment (30 ng/ml for 5 min) enhanced ASIC currents by stimulated by three different acidic solutions at pH 6.5, pH 5.5, and pH 4.5. *B*, the graph shows the concentration-response curves for acidic stimuli with and without pre-application of IL-33 (30 ng/ml). IL-33 treatment shifted upwards the concentration-response curve for acidic stimuli, showing a significant increase in the normalized maximum response to acidic stimuli. The curves were drawn according to the logistic equation I = I _max_/[1 + (10ˆ-pH_0.5_/10ˆ-pH) ^n^], where I is the normalized current response value, pH_0.5_ is the pH for half-maximal activation, and n is the Hill coefficient. All current values from the same cell were normalized to the current response to pH 4.5 acidic stimuli alone (marked with an *asterisk*). Each point represents the mean ± SD of 7 to 9 cells from 5 to 7 mice.

We also investigated the effect of IL-33 on ASIC currents evoked by different acidic stimuli. The current traces in Figure 2*A* show that IL-33 pretreatment (30 ng/ml for 5 min) had an enhancing effect on the three ASIC currents induced by acidic stimuli at pH 6.5, pH 5.5, and pH 4.5, respectively. Figure 2*B* shows that IL-33 pretreatment shifted upwards the concentration-response curve for acidic pH. IL-33 had a 43.1 ± 14.3% enhancing effect on the normalized maximal current response (I_pH4.5_) that was induced by an acidic stimulus at pH 4.5 (*p* < 0.01, two-way repeated measures ANOVA followed by Bonferroni *post hoc* correction, n = 7). In contrast, there was no significant difference in the pH_0.5_ (pH for half-maximal activation) values between the two curves (pH: pH_0.5_ = 5.7 ± 0.7; IL-33 + pH: pH_0.5_ = 5.9 ± 0.9). Il-33 did not affect the Hill coefficient of the curve (pH: n = 1.4 ± 0.3; IL-33 + pH: n = 1.6 ± 0.5). These results indicated that IL-33 increased the ASIC's maximum responses to acidic stimuli but did not alter its sensitivity.

### ST2 mediates the enhancement of ASIC currents by IL-33

**Figure 3:**
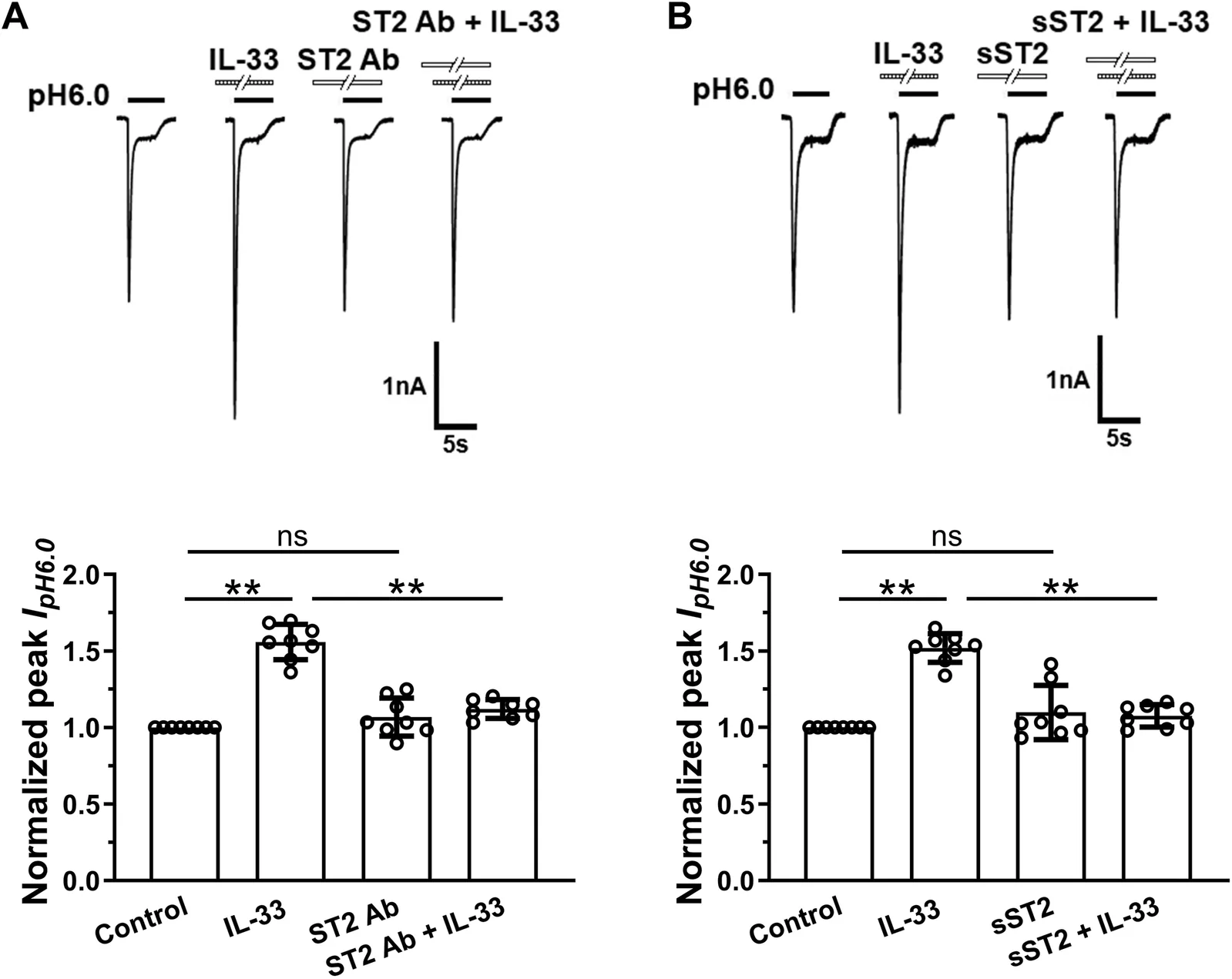
**Participation of ST2 in the IL-33-induced potentiation of ASIC currents.** Representative current traces and the bar graph in (*A*) show that the specific ST2 neutralizing antibody (ST2Ab, 2 μg/ml) abrogated the IL-33-induced potentiation of I_pH6.0_. ST2Ab alone had no effect on the amplitude of the peak I_pH6.0_. Representative current traces and the bar graph in (*B*) show that the IL-33-induced potentiation of ASIC currents was prevented by co-treatment with the decoy receptor for IL-33 (sST2, 300 nM). ∗∗*p* < 0.01, ns, not significant; one-way ANOVA followed by Tukey's *post hoc* test, n = 8 cells from 5 mice in each column.

It has been shown that IL-33 fulfills a variety of biological functions through the membrane ST2 (1, 4). Thus, we determined whether ST2 participated in the IL-33-induced enhancement of ASIC currents. First, DRG neurons were simultaneously treated with IL-33 and a specific ST2 neutralizing antibody (ST2Ab). Representative current traces and the mean summary data in Figure 3*A* show that the I_pH6.0_ increased by 56.3 ± 11.7% after the application of IL-33 (30 ng/ml) alone, while co-treating DRG neurons with the ST2Ab (2 μg/ml) significantly abolished the enhancement in the I_pH6.0_ amplitude induced by IL-33 (12.1 ± 6.1% of the control, *p* < 0.01, paired *t* test, n = 8). Figure 3*B* show that the IL-33-induced enhancement of I_pH6.0_ was prevented by co-treatment with the decoy receptor for IL-33 (sST2, 300 nM; 7.6 ± 7.2% of the control, n = 8). In addition, application of ST2Ab alone or sST2 alone had no significant effect on ASIC currents (Fig. 3, *A* and *B*). These results indicated the participation of ST2 in the IL-33-induced enhancement of ASIC currents.

### p38 MAPK is involved in the IL-33-mediated enhancement of ASIC currents

**Figure 4:**
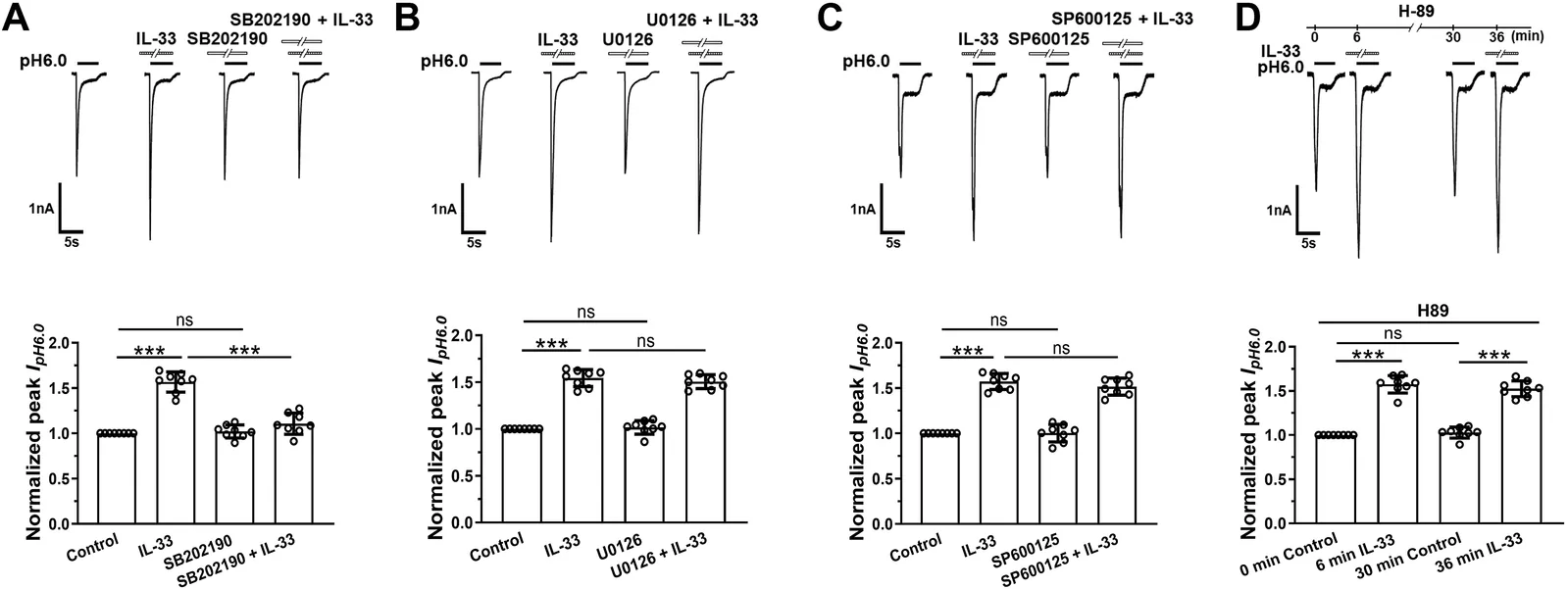
**Involvement of p38 MAPK in the IL-33-mediated enhancement of ASIC currents.** The current traces and the bar graph in (*A*-*C*) show changes of I_pH6.0_ amplitude induced by IL-33 in cells pretreated with 10 μM SB202190, 10 μM U0126, 10 μM or SP600125. The cells pretreated with SB203580 prevented the IL-33-mediated enhancement of I_pH6.0_ amplitude, whereas the enhancing effects of IL-33 on I_pH6.0_ amplitude were not affected by pretreatment with U0126 and SP600125. SB202190, U0126 and SP600125 *per se* had no effect on I_pH6.0_ amplitude. The current traces and the bar graph in (*D*) show that IL-33 enhanced I_pH6.0_ when applied 6 min and 36 min after establishing the whole-cell configuration in the presence of 2 μM H-89. ∗∗*p* < 0.01, ns, not significant; one-way ANOVA followed by Tukey's *post hoc* test. n = 8 cells from 5 to 7 mice in each column.

To determine the signaling pathways facilitating the IL-33-mediated enhancement of ASIC currents, we examined whether pretreatment of DRG cells with selective signaling pathway inhibitors could delineate the intracellular signaling mechanisms underlying the IL-33-mediated responses. The MAPK signaling molecules were examined. We pre-incubated DRG cells with the p38 MAPK inhibitor SB202190, the ERK1/2 inhibitor U0126, or the JNK inhibitor SP600125. Unlike an enhancement of the IpH6.0 amplitude by IL-33 alone, the IL-33-induced increase in the IpH6.0 amplitude was significantly reduced in the presence of 10 μM SB202190 (10.7 ± 11.7% *versus* 56.7 ± 11.2%; *p* < 0.001, paired *t* test, n = 8; Fig. 4*A*). Similar to the enhancement by IL-33 alone, IL-33 showed a comparable level of increase for I_pH6.0_ in the presence of 10 μM U0126 (50.6 ± 7.4% *versus* 54.4 ± 9.1%; Fig. 4*B*) or 10 μM SP600125 (51.6 ± 9.4% *versus* 57.3 ± 8.8%; Fig. 4*C*). In addition, SB202190, U0126 and SP600125 *per se* had no effect on I_pH6.0_ amplitude. The protein kinase A (PKA) inhibitor H-89 was also added to the intracellular solution. Figure 4*D* shows that IL-33 had a similar enhancing effect on the I_pH6.0_ when applied 6 min and 36 min after establishing the whole-cell configuration in the presence absence of 2 μM H-89 (52.5 ± 8.9% *versus* 57.5 ± 9.9%). Taken together, these results indicated that IL-33-mediated enhancement of ASIC currents requires intracellular p38 MAPK but not ERK1/2, JNK, and PKA.

### IL-33 potentiates DRG neuronal excitability triggered by acidic stimuli

**Figure 5:**
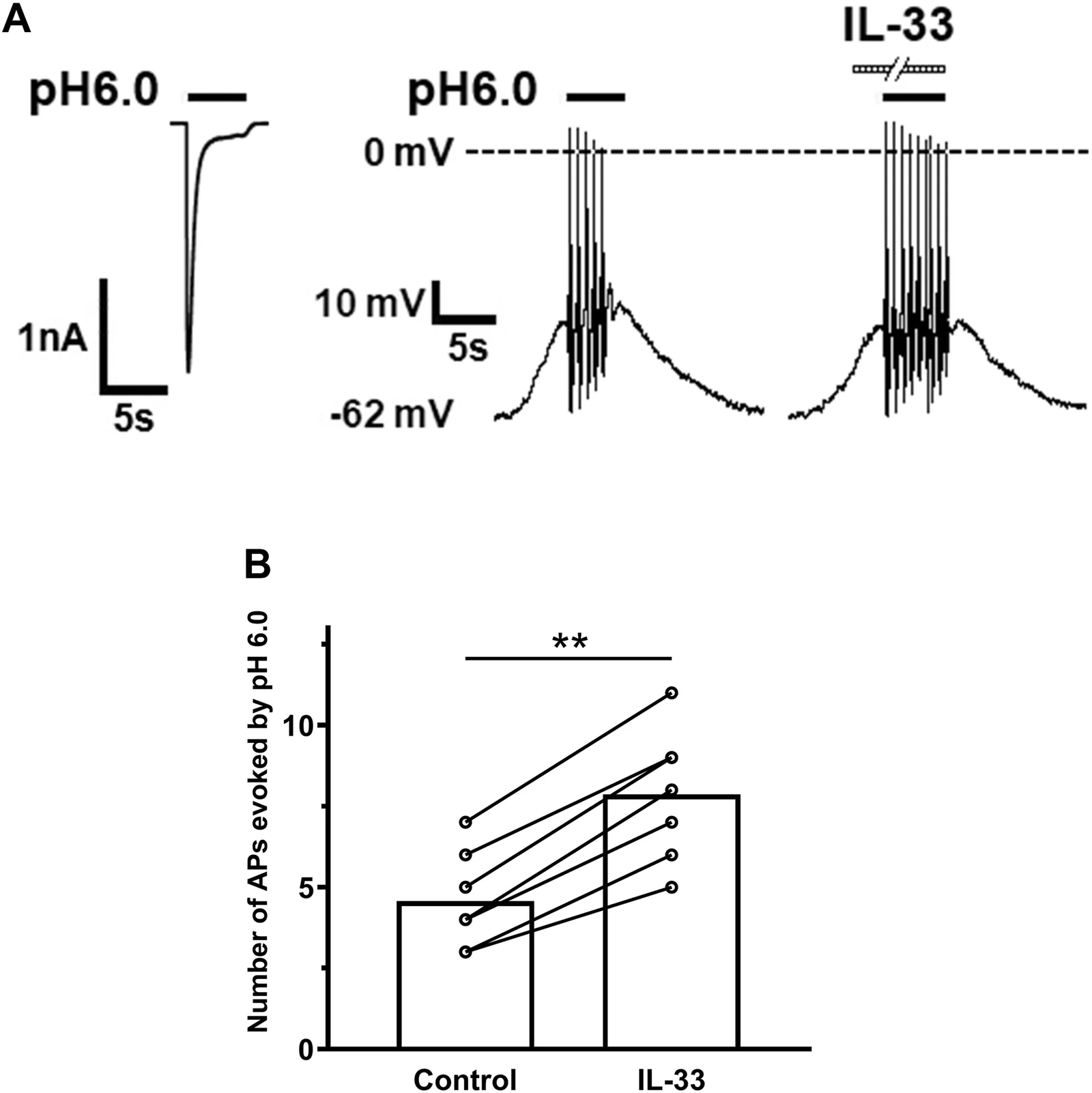
**Potentiation of acid-triggered action potentials by IL-33 in DRG neurons.** The original traces in (*A*) show that an acidic stimulus at pH 6.0 caused an inward current under voltage-clamp conditions, and also triggered the bursts of action potentials (APs) under current-clamp conditions in the same DRG cell. The number of acidic stimulus-triggered APs was increased after the cell was treated with 30 ng/ml IL-33 for 5 min. The bar graph in (*B*) summarizes the mean number of APs triggered by acidic stimuli under control and IL-33-treated conditions. ∗∗*p* < 0.01, paired *t* test; n = 7 cells from 5 mice.

Further, we studied whether IL-33 affects action potentials (APs) evoked by acidic stimuli in DRG neurons. Figure 5*A* shows that an acidic stimulus at pH 6.0 evoked a large inward current and a burst of APs in the same cell under voltage- and current-clamp conditions, even in the presence of 5 μM AMG9810. Consistent with its enhancing effect on ASIC currents, IL-33 also potentiated the bursts of acid-evoked APs under current-clamp conditions. Representative trace samples in Figure 5*A* show that the same acidic stimulus triggered a greater number of APs in the cell pretreated with 30 ng/ml IL-33 for 5 min. The mean summary data in Figure 5*B* show that the number of APs evoked by acidic stimuli at pH 6.0 increased from 4.6 ± 1.5 in the control conditions to 7.9 ± 2.0 in the IL-33-treated conditions (*p* < 0.01, paired *t* test; n = 7 cells from 5 mice). The results indicated that IL-33 also potentiated the excitability of DRG neurons to acidic stimuli.

### Contribution of ASIC3 to IL-33-induced mechanical hyperalgesia *in vivo*

**Figure 6:**
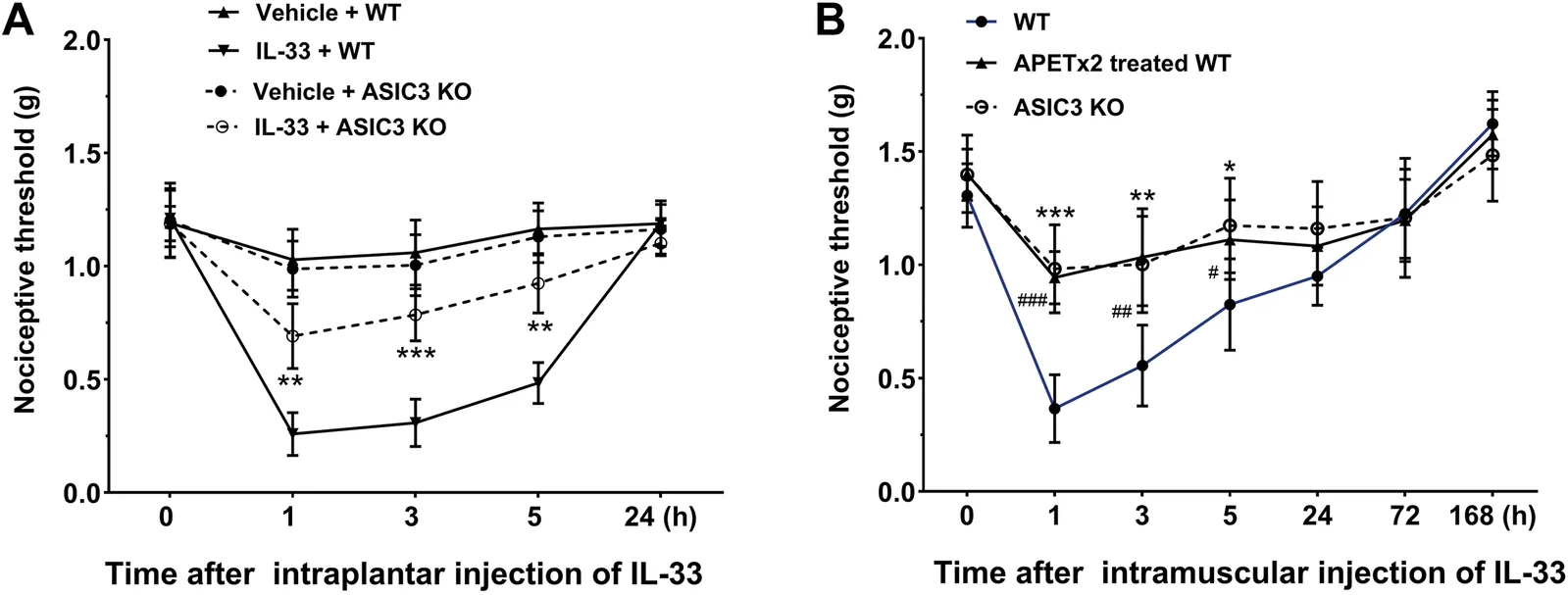
**Involvement of ASIC3 in IL-33-induced mechanical hyperalgesia *in* vivo.***A*, time course of mechanical hyperalgesia induced by intraplantar injection of IL-33. There was a significant difference in the changes of paw withdrawal threshold (PWT) induced by intraplantar injection of IL-33 (50 ng in 25 μl) between WT and ASIC3 KO mice (F _(4)_ = 145.8; *p* < 0.0001, factor genotype × time). Compared with vehicle injection, IL-33 significantly decreased the PWT in WT mice, but this effect was attenuated in ASIC3 KO mice. ∗∗*p* < 0.01, ∗∗∗*p* < 0.001, IL-33-treated WT mice *versus* IL-33-treated ASIC3 KO mice at the same time point, two-way ANOVA with a Bonferroni *post hoc* test. Each point represents the mean ± SD of 10 mice. *B*, time course of mechanical hyperalgesia induced by intramuscular injection of IL-33 (50 ng in 25 μl). Repeated measures two-way ANOVA showed significant effects for genotype treatment (F _(6)_ = 103.8; *p* < 0.0001, factor genotype × time) and drug (F _(6)_ = 83.4; *p* < 0.0001, factor drug × time). Injection of IL-33 into the gastrocnemius muscle produced a significant decrease in mechanical nociceptive threshold in WT mice, but the effect was attenuated in ASIC3 KO mice and WT mice treated with the ASIC3 blocker APETx2. APETx2 (228 ng, 25 μl of 2 μM APETx2 solution) was treated into the gastrocnemius muscle together with IL-33. ∗*p* < 0.05, ∗∗*p* < 0.01, ∗∗∗*p* < 0.001, ASIC3 KO mice *versus* WT mice, #*p* < 0.05, ##*p* < 0.01, ###*p* < 0.001, APETx2-treated WT mice *versus* WT mice, at the corresponding time points; two-way ANOVA with a Bonferroni *post hoc* test. Each point represents the mean ± SD of 10 mice.

Finally, we investigated whether ASIC3 are involved in IL-33-induced pain sensitivity *in vivo*. IL-33-induced mechanical hyperalgesia was examined in ASIC3 KO mice and their WT littermates. Compared with vehicle injection, Figure 6*A* shows that intraplantar injection of IL-33 (50 ng in 25 μl) induced significant decreases in the paw withdrawal thresholds (PWT) in WT mice, indicating a significant increase in sensitivity to mechanical stimuli. However, IL-33 evoked attenuated mechanical hyperalgesia in ASIC3 KO mice, as shown by a weakened decrease in PWT after intraplantar injection of 50 ng of IL-33 (Fig. 6*A*). Intramuscular injection of IL-33 (50 ng in 25 μl) also produced mechanical hyperalgesia. After IL-33 was injected into the gastrocnemius muscle, it produced a decrease in mechanical nociceptive threshold in WT mice, peaking about 1 h and persisting for at least 5 h after injection (Fig. 6*B*). In contrast, after L-33 was injected into the gastrocnemius muscle, ASIC3 KO mice displayed significantly attenuated mechanical hyperalgesia compared to wild-type mice (Fig. 6*B*). In addition, the IL-33-induced mechanical hyperalgesia in WT mice was significantly attenuated by intramuscular co-injection of the ASIC3 blocker APETx2 (228 ng, 25 μl of 2 μM APETx2 solution; Fig. 6*B*). The results indicated that ASIC3-deficient mice displayed attenuated mechanical hyperalgesia induced by intraplantar or intramuscular injection of IL-33.

## Discussion

**Figure 7:**
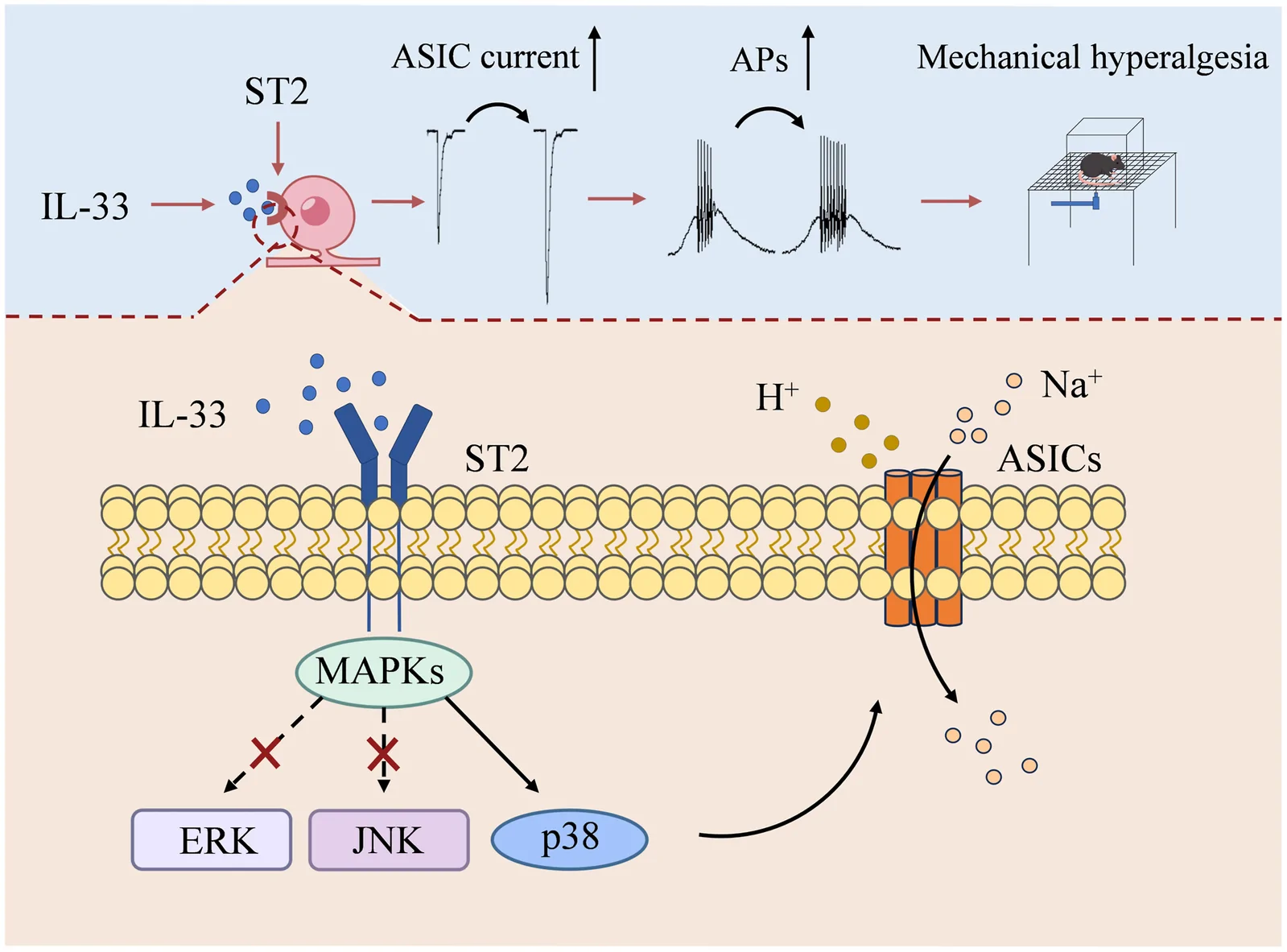
**Schematic showing the molecular mechanism of IL-33-induced enhancement of ASIC currents in mouse DRG neurons.** IL-33 binds to the transmembrane ST2 receptor to stimulate the downstream p38 MAPK signaling pathway, which in turn regulates ASICs activity. IL-33/ST2-mediated signaling enhances acid-induced ASIC currents and APs, leading to mechanical hyperalgesia. Neither ERK nor JNK was necessary for the IL-33-induced enhancement of ASIC currents.

The present data demonstrated that there was a functional link between IL-33/ST2 signaling and ASICs in peripheral nociceptive processing. IL-33 enhanced ASIC function through ST2. IL-33 increased acid-activated ASIC currents and APs in dissociated mouse DRG neurons *via* p38 MAPK-dependent signaling (see Fig. 7). Behaviorally, the mechanical hyperalgesia induced by IL-33 was attenuated in ASIC3-deficient mice.

ASIC3 was initially cloned from rodent sensory neurons and has consistently been found predominantly in DRG neurons (23, 37, 38). In the present study, the acid-induced currents were blocked by the ASIC channel blocker amiloride and by the ASIC3 blocker APETx2, indicating ASIC3-like currents that were mediated by ASIC3 homomeric or ASIC3-containing heteromeric channels (39). We observed that the Hill coefficient for the ASIC3-like current activation in DRG neurons was lower than the slope or Hill coefficient of 2.36 in homomeric ASIC3-expressing CHO cells (40). One explanation may be that the present ASIC3-like currents were due to activation of ASIC3-containing heteromeric channels rather than ASIC3 homotrimers. This was consistent with the morphological evidence that ASICs are expressed in DRG neurons, with ASIC3 being the most prevalent ASIC subunit (23, 24, 33). Therefore, the acid-induced electrophysiological recordings was consistent with the expression of ASIC3 in DRG neurons. We found that the ASIC3-like currents were increased in these recorded DRG neurons pretreated with IL-33. IL-33 treatment dose dependently increased the peak amplitude of ASIC3-like currents. IL-33 also increased the maximum response of ASICs to acidic stimuli but did not affect their sensitivity. Under current-clamp conditions, IL-33 treatment potentiated DRG neuronal excitability, showing an increased number of acid-triggered APs. These findings that IL-33 enhanced the acid-induced electrophysiological activity in mouse DRG neurons indicated that ASICs served as downstream modulatory targets of IL-33 signaling.

How did IL-33 enhance ASICs? Immunostaining of ST2 on DRG has demonstrated that ST2 is expressed in CGRP-labeled peptidergic neurons, IB4-labelled nonpeptidergic neurons, and NF200-positive myelinated neurons (8, 17). In the present study, ST2 did appear to be involved in the IL-33-induced enhancement of ASIC currents, since pretreating DRG neurons with a specific ST2Ab completely abolished the increase in ASIC currents induced by IL-33. In addition, the effect of IL-33 on ASIC currents was also prevented by sST2, the decoy receptor for IL-33. Therefore, IL-33 may exert a direct action on mouse DRG neurons through ST2 localized on the neuronal membrane. In the present study, only some, but not all, ASIC3-like responses were enhanced by IL-33, which may occur only in ST2-expressing DRG neurons, although further morphological evidence is needed. It has been shown that IL-33 inhibits type A potassium channels in mouse DRG neurons by activating ST2 (17).

Did IL-33/ST2 act directly or indirectly on ASICs? Studies have shown that IL-33 contributes to pathological pain through NF-κB, MAPKs, JAK2/STAT3, and/or PI3K/AKT signaling pathways (10, 41, 42). Among these signaling pathways, the members of the MAPK family, including extracellular p38, ERK, and JNK, play critical roles in regulating pain responses (43). MAPKs have been shown to modulate ASIC currents. For example, pharmacological inhibition of ERK prevents the enhancing effect of hydrogen sulfide on ASIC currents, indicating the involvement of ERK signaling (44). Activating JNK can enhance the activity of ASIC1b- and ASIC3-containing channels within minutes (45). Our previous study shows that rapid enhancement of ASIC currents by tumor necrosis factor-α depends on p38 MAPK signaling in rat DRG neurons (46). However, it remains unclear whether MAPK signaling participates in the enhancement of ASIC currents by IL-33/ST2 in mouse DRG neurons. Thus, we had detected the involvement of MAPKs in the IL-33-mediated enhancement of ASIC currents by incubating DRG cells with the p38 MAPK inhibitor SB202190, the ERK inhibitor U0126, or the JNK inhibitor SP600125. The present data showed that the IL-33-induced enhancement of ASIC currents was significantly abolished by SB202190, but not by U0126 and SP600125, indicating intracellular p38 MAPK signaling, rather than ERK and JNK, was required for the effects of IL-33/ST2 on ASICs. Immunostaining reveals that p38 MAPK is co-expressed with ST2 in mouse DRGs neurons, and ST2 stimulation with IL-33 elevates p-p38 levels in mouse DRG cells, whereas p-ERK and p-JNK remains unaffected (17). Further, the study shows that the activation of Syk is required for the increase of p-p38 levels by IL-33 (17). It has been demonstrated that Syk can also regulate PKA activity that might participate in the regulation of ASIC currents (47, 48). However, IL-33-induced enhancement of ASIC currents was not affected in DRG neurons pretreating the PKA-specific inhibitor H-89. The result excludes intracellular PKA signaling in the IL-33/ST2-mediated enhancement. Together, we speculate that IL-33/ST2 acted indirectly on ASICs in mouse DRG neurons *via* the intracellular p-p38 MAPK signaling pathway, resulting in increased ASIC currents induced by acid. However, whether p-p38 MAPK directly phosphorylates ASICs or stimulates intermediate molecules in mouse DRG neurons needs to be investigated further.

Studies have shown that IL-33 elicits mechanical hyperalgesia (15, 49). The present data showed that intraplantar and intramuscular injection of IL-33 produced a time-dependent mechanical hyperalgesia in WT mice, consistent with its reported pro-algesic effect after intra-articular or cutaneous administration (11, 15, 49). Our findings indicated that ASIC3 contributed to IL-33-induced mechanical hyperalgesia, since the IL-33-induced hyperalgesia was significantly reduced in ASIC3-deficient mice. The mechanical hyperalgesia induced by intramuscular injection of IL-33 was also prevented by APETx2 treatment in WT mice, indicating the involvement of ASIC3. Since APETx2 at 2 μM concentration can also inhibit Nav 1.8 channels, therefore, we cannot rule out that the effect on APETx2 on IL-33 induced mechanical hyperalgesia was driven by Nav 1.8 channels, rather than ASICs alone (50). Acidic stimuli activate ASIC3 channels, causing the influx of Na^+^ ions, subsequently membrane depolarization, which is sufficient to trigger bursts of APs in DRG neurons (27). The APs triggered by acidic stimuli are transmitted to the brain to produce nociception. Under current-clamp conditions, IL-33 also enhanced the membrane excitability of mouse DRG neurons, showing the increased number of acid-triggered APs in the presence of IL-33. ASIC3 is expressed in about 50% of small muscle afferents (51), which increases during muscle inflammation (35). Therefore, as a downstream molecule of IL-33, it is not surprising that genetic deletion of ASIC3 can ameliorate mechanical hyperalgesia induced by intramuscular injection of IL-33. IL-33/ST2 signaling sensitized ASIC3 on primary afferents, which underlined IL-33-induced mechanical hyperalgesia. Of course, the present data did not exclude the possibility that other mechanisms underlined IL-33-induced mechanical hyperalgesia, such as modulation of voltage-gated channels by IL-33/ST2 signaling (17).

What is the physiological importance of ASIC modulation by IL-33/ST2 signaling? Tissue acidification commonly develops in various painful conditions such as inflammation, nerve injury, ischemia, fatiguing muscle, and tumors (52, 53). ASICs, especially ASIC3, localized on peripheral terminals, can detect pH drops and respond to acidosis (33). After several types of cellular stress, including inflammation, tissue injury, ischemia, and mechanical strain, IL-33 is released and is detected by ST2 located on nociceptors and subsequently triggers mechanical hyperalgesia (8, 54, 55, 56). For example, ST2-positive fibers are detected in the skin, and levels of IL-33 in the skin increase during inflammatory pain (7, 8). Therefore, it is conceivable that IL-33 produced hyperalgesia through ST2-positive fibers in the skin. Since both IL-33 and protons can be released under pathological pain conditions, they can sensitize and/or initiate the nociceptive process by activating their cognate receptors and channels located on the peripheral terminals of the nociceptive neurons. Once both IL-33 and protons are released locally in the same painful area, the present results showed a functional link between IL-33/ST2 signaling and ASICs. IL-33 could sensitize ASICs on peripheral nerve endings, amplifying acid-induced responses. IL-33 initiated mechanical hyperalgesia that required ASIC3. Knocking out the downstream ASIC3 can weaken the roles of IL-33 in the pain process, as observed with the attenuated mechanical hyperalgesia caused by IL-33 in ASIC3-deficient mice.

In summary, our results indicated that ASICs, particularly ASIC3, are downstream targets of IL-33/ST2 signaling. ST2 stimulation by IL-33 enhanced ASIC-mediated electrophysiological activity in primary sensory neurons *via* the intracellular p38 MAPK signaling pathway, which contributed to IL-33-induced mechanical hyperalgesia.

## Experimental procedures

### Preparation and culture of DRG neurons

The experimental protocol was approved by the Hubei University of Science and Technology's Animal Research Ethics Committee (2016-03-005). Male and female mice (C57BL/6, 6–8 weeks old) were anesthetized and then sacrificed by decapitation. DRGs from the fourth to sixth segments of the lumbar were collected and chopped. The ganglia were transferred to a tube containing Dulbecco's Modified Eagle Medium (DMEM) and digested with collagenase (type I-A, 1.0 mg/ml), trypsin (type II-S, 0.5 mg/ml), and DNase (type IV, 0.1 mg/ml) for about 20 min at 36 °C. A soybean trypsin blocker (type II-S, 1.25 mg/ml) was used to terminate the digestion. Dissociated DRG cells were placed into a 35 mm Petri dish and cultured for 12 to 24 h at 37 °C in DMEM containing 100 ng/ml nerve growth factor and 10% fetal bovine serum before electrophysiological recordings.

### Electrophysiological recordings

Electrophysiological experiments were performed as described previously (57, 58). Currents and action potentials were recorded using a MultiClamp-700B amplifier (Axon Instruments). Before electrophysiological recordings, the cultured DRG cells were kept in a 35 mm plate filled with an external solution (in mM): 150 NaCl, 2.5 CaCl_2_, 2 MgCl_2_, 5 KCl, 10 D-glucose, and 10 HEPES, adjusted to pH 7.4 with NaOH. The patch pipettes were filled with a solution containing (in mM): 140 KCl, 2 MgCl_2_, 0.3Na_2_GTP, 4 Na_2_ATP, 11 EGTA, and 10 HEPES, adjusted to pH 7.4 with KOH. After gigaseal formation, the current transients were compensated, and the membrane was ruptured, forming a whole-cell recording configuration. Series resistance was compensated by 70 to 80%. The currents were recorded with a 10 kHz sample rate and a 2 kHz low-pass filter. All recordings were only performed on small- and medium-sized nociceptive DRG cells (15–40 μm in diameter) that have been identified as nociceptive neurons (59). Current-clamp recordings were performed only on DRG cells with resting membrane potentials less than −50 mV.

### Drug application

In the experiment, all chemicals were purchased from commercial suppliers. Hydrochloric acid was sourced from Beyotime. IL-33, ST2Ab, sST2, SB202190, U0126, SP600125, H-89, amiloride, APETx2, capsaicin, AMG 9810, all enzymes and enzyme inhibitors for DRG cell isolation were purchased from Sigma-Aldrich (Sigma-Aldrich). All working drugs were prepared with the external solution. Hydrochloric acid was adjusted to the corresponding pH values using NaOH (1 mM) and MES (10 mM), while other working drugs were adjusted to7.4 using NaOH (1 mM). Each working drug was stored in a series of independent reservoirs and applied by gravity. The distance was about 30 μm between the drug exit and the recorded neurons. After extracellular application of the working drugs, the recorded DRG cells were washed with the external solution for 5 min. To purify the ASIC currents, we recorded currents in the extracellular solution containing the TRPV1 blocker AMG9810 (5 μM). In DRG cells, ASIC currents were recorded at 6-min intervals, and the cells were washed with external solution for 1 min before IL-33 treatment for 5 min. If an inhibitor was used, DRG cells were perfused with external solution containing the inhibitor for 1 min, followed immediately by treatment with a mixture of the inhibitor and IL-33 for 5 min. After the inhibitor and IL-33 were mixed, they were immediately stored in an independent reservoir for perfusion DRG cells. To modify intracellular signaling, membrane-permeable inhibitor SB202190, U0126 or SP600125 was introduced to an external solution. H-89 was dissolved in the intracellular solution, and it entered the recorded cells through the membrane patch pores between the recording patch pipettes and the sealed cells in a molecular diffusion manner. As described previously, H-89 and other dialysis drugs can fully diffuse into the cell about 25 min after the cell membrane ruptures (40). To ensure that the cell interior was perfused with H-89, we tested the effect of IL-33 on ASIC currents twice, with at least a 30-min interval between the tests after establishing the whole-cell configuration (40).

### Measurement of hyperalgesia

All behavioral measurements were performed using littermate WT and ASIC3 KO mice (6–8 weeks old, C57BL/6 background). Paw withdrawal thresholds (PWTs) and mechanical nociceptive threshold in the gastrocnemius muscle were tested using the up and down method (60). A series of von Frey filaments (Stoelting) were used to vertically press against the injected hind paw or the gastrocnemius muscle until the filaments were bent for 6 s. Quick withdrawal was counted as a positive response. The test was carried out many times in each mouse, and the interval between tests was at least 5 min. Behavioral experiments were performed in a blinded manner. The main purpose of the behavioral experiments in this study was to compare the genotype differences of IL-33 effects in ASIC3 KO mice and their WT littermates in the two pain models. Therefore, we only designed vehicle injection as a control in Figure 6*A*, but not in Figure 6*B*.

### Data analysis

The means ± SD were used for all data. The normality of the data distribution and the homogeneity test of variance were initially performed. A paired *t* test is used to compare differences pre- and post-treatment in the same group of cells. Multiple comparisons were performed using one-way and repeated measures analysis of variance (ANOVA) followed by Tukey's *post hoc* test. Bivariate parametric analysis was performed using two-way repeated measures ANOVA followed by Bonferroni's *post hoc* test. The nonlinear curve fitting tool ALLFIT was used to perform statistical analysis on the concentration-response data.

## Ethics statement

The experimental protocol was approved by the Hubei University of Science and Technology's Animal Research Ethics Committee (2016-03-005).

## Data availability

All data and analyses are included in the main text of the article.

## Conflict of interest

The authors declare that they have no conflicts of interest with the contents of this article.
